# Association between cardiorespiratory fitness and body fat in girls

**DOI:** 10.1016/j.rppede.2016.02.014

**Published:** 2016

**Authors:** Giseli Minatto, Thiago Ferreira de Sousa, Wellington Roberto Gomes de Carvalho, Roberto Régis Ribeiro, Keila Donassolo Santos, Edio Luiz Petroski

**Affiliations:** a Centro de Desportos, Núcleo de Pesquisa em Cineantropometria e Desempenho Humano, Universidade Federal de Santa Catarina (UFSC/CDS/NuCiDH), Florianópolis, SC, Brazil; b Universidade Federal do Triângulo Mineiro, Uberaba, MG, Brazil; c Laboratório de Estudos e Pesquisas Epidemiológicas em Atividade Física, Exercício e Esporte (LAPAES), Departamento de Educação Física, Universidade Federal do Maranhão, São Luís, MA, Brazil; d Faculdade Assis Gurgacz, Cascavel, PR, Brazil

**Keywords:** Adolescent, Physical fitness, Body composition

## Abstract

**Objective:**

To estimate the prevalence of low cardiorespiratory fitness and its association with excess body fat, considering the sexual maturation and economic level in female adolescents.

**Methods:**

Cross-sectional, epidemiological study of 1223 adolescents (10-17 years) from the public school system of Cascavel, PR, Brazil, in 2006. We analyzed the self-assessed sexual maturation level (prepubertal, pubertal and post-pubertal), the economic level (high and low) through a questionnaire and body fat (normal and high) through triceps and subscapular skinfolds. The 20-meter back-and-forth test was applied to estimate maximum oxygen consumption. Cardiorespiratory fitness was assessed according to reference criteria and considered low when the minimum health criterion for age and sex was not met. Chi-square test and logistic regression were applied, with a significance level of 5%.

**Results:**

The prevalence of low cardiorespiratory fitness was 51.3%, being associated with all study variables (*p*<0.001). At the crude analysis, adolescents with high body fat were associated with low cardiorespiratory fitness, when compared to those with normal body fat (*OR*=2.76; 95%CI: 2.17-3.52). After adjustment by sexual maturation, this association remained valid and showed an effect that was 1.8-fold higher (95%CI: 1.39-2.46) and after adjusting by economic level, the effect was 1.9-fold higher (95%CI: 1.45-2.61).

**Conclusions:**

Approximately half of the assessed girls showed unsatisfactory levels of cardiorespiratory fitness for health, which was associated with high body fat, regardless of sexual maturation level and economic level. Effective public health measures are needed, with particular attention to high-risk groups.

## Resumo

**Objetivo:**

Estimar a prevalência de aptidão cardiorrespiratória baixa e sua associação com excesso de adiposidade corporal, considerando a maturação sexual e o nível econômico, em adolescentes do sexo feminino.

**Métodos:**

Estudo epidemiológico transversal com 1.223 adolescentes (10-17 anos) da rede pública de ensino de Cascavel, PR, Brasil, em 2006. Analisou-se a maturação sexual (pré-púbere, púbere e pós-púbere) autoavaliada, o nível econômico (NE) (alto e baixo) por questionário e a adiposidade corporal (normal e elevada) por dobras cutâneas do tríceps e subescapular. Aplicou-se o teste de vaivém de 20 metros para estimar o consumo máximo de oxigênio. A aptidão cardiorrespiratória foi avaliada por critérios referenciados e considerada baixa quando não atingido o critério mínimo para a saúde segundo idade e sexo. Foram aplicados o teste de qui-quadrado e a regressão logística, com nível de significância de 5%.

**Resultados:**

A prevalência de aptidão cardiorrespiratória baixa foi de 51,3% que se associou a todas as variáveis do estudo (*p*<0,001). Na análise bruta, as adolescentes com adiposidade corporal elevada associaram-se à aptidão cardiorrespiratória baixa, quando comparada com aquelas com adiposidade normal (RC=2,76; IC95% 2,17-3,52). Após ajuste pela maturação sexual, essa associação se manteve e mostrou efeito 1,8 vez maior (IC95% 1,39-2,46) e, após ajuste pelo NE, o efeito foi 1,9 vezes maior (IC95% 1,45-2,61).

**Conclusões:**

Aproximadamente metade dos avaliados apresentou níveis insatisfatórios de aptidão cardiorrespiratória para a saúde, o que se associou à adiposidade corporal elevada, independentemente da maturação sexual e NE. Medidas efetivas de saúde pública são necessárias, com especial atenção para grupos de maior risco.

## Introduction

Cardiorespiratory fitness is considered an important marker of health since childhood and adolescence.^1^ It is defined as the capacity of the circulatory and respiratory systems to supply oxygen to the muscles during exercise of moderate to high intensity and involves large muscle groups for long periods of time.^2^

Studies indicate that cardiorespiratory fitness in children and adolescents has decreased in recent decades in 27 countries (reduction of 0.46%)^3^ and in Brazil (reduction of 0.51%).^4^ The proportion of adolescents that do not reach acceptable levels of this component of physical fitness for health varies from 37.8%^5^ in Florianopolis to 60%^6^ in Parana.

The low level of cardiorespiratory fitness is associated with increased cardiovascular risk factors and metabolic syndrome in young individuals,^7^ as well as to increased cardiovascular risk in adulthood.^8^ A meta-analysis showed that the overall risk of death from all causes or from cardiovascular disease was two-fold higher in individuals with low cardiorespiratory fitness levels, compared to those with high levels.^8^ According to Azambuja et al.,^9^ in 2004 the annual cost to public funds for the treatment of cardiovascular disease was R$ 30.8 billion (36.4% for health, 8.4% for social security and reimbursement by employers and 55.2% as a result of lost productivity).

Another aggravating factor of low fitness levels is excess body fat even at early ages. Adolescents with excess body fat have lower cardiorespiratory fitness,^6^^,^ 10 ^,^ 11 predominantly girls.^10^ The increased gain in adiposity in adolescence is associated with the onset of puberty, at which stage girls, through action of the hormone estrogen, tend to accumulate higher amounts of body fat.^12^ However, there is a study that reports a negative association between cardiorespiratory fitness and sexual maturation, which controlled the percentage of body fat in girls^10^ and may indicate changes in cardiorespiratory fitness during the maturational and physical development.

Although the association between cardiorespiratory fitness and body adiposity has been explored in adolescents,^6^^,^ 13 there is a gap in the literature on the association between these variables while considering the economic level. In adolescents of high economic level, better cardiorespiratory fitness was found in adolescents with less accumulation of body fat.^6^

When only the boys are assessed, the association between body fat and other physical fitness components is also inverse, but seems to differ between social strata.^13^ Other authors, in a study involving both genders, reported no association between cardiorespiratory fitness and economic level.^5^ These conflicting results emphasize the need for further studies to elucidate the association between cardiorespiratory fitness and body fat in different economic strata, as preventive actions might consider such aspects. Therefore, this study aimed to estimate the prevalence of low cardiorespiratory fitness and its association with excess body fat, while considering sexual maturation and the economic level in female adolescents (aged 10-17 years) from the city of Cascavel, Paraná state, Brazil.

## Method

This is a school-based epidemiological and cross-sectional study carried out in 2006 in Cascavel city, West region of Paraná state, Southern Brazil. The estimated population of the municipality in 2006 was of 245,369 inhabitants, with the majority (93.2%) living in the urban area.^14^ The Human Development Index (HDI) of the municipality is 0.810 (high human development).^15^

This article is part of a larger epidemiological, cross-sectional study, called “Anthropometry, body composition, motor performance and sexual maturation of students from different socioeconomic levels in Cascavel city, Paraná”. The target population of the largest study consisted of female students aged 8-17 years, living in the urban area.

According to the report of the Regional Education Center and the Municipal Education Secretariat of Cascavel, in 2006 the municipality had 39,830 students enrolled in elementary and high schools. Given that the Municipal Secretariat of Education does not provide the number of students by gender, a distribution of 50% was considered, totaling a population of 19,915 female students. The sample calculation followed the procedures suggested by Barbetta,^16^ with an expected prevalence of 50% for the outcome, sample error of 2 percentage points and 95% confidence interval, resulting in a sample of 2221 girls.

The sampling procedure consisted of a three-stage conglomerate, with the first being the educational district, the second being the school and the third the classes, respecting the proportionality. Three educational districts were formed according to the distribution of students in different geographic regions of the municipality in order to ensure better representativeness, according to the geographical division proposed by the Regional Education Center of Cascavel, PR. Student distribution showed a ratio of 35.8% at district I; 33.1% in district II and 31.2% in district III.

At the first stage, four schools were chosen from each district by drawing lots, two municipal and two state schools. In the second stage, the schools that would participate in the study were chosen from a list provided by the institutions themselves, which contained to the students' ages, by drawing lots. At the third stage, we carried out a simple random selection of classes, considering the proportionality in relation to the target population.

For this study, the calculation of the sample's statistical power was carried out retrospectively. To test the association between body fat (exposure) and cardiorespiratory fitness (outcome), a prevalence of exposure of 38%, a prevalence of outcome in the unexposed of 42% and a confidence level of 95% were considered with the analyzed sample (*n*=1223). The study had 100% power to find an odds ratio of 1.6 or higher as significant.

For the present study, only the adolescents (10-17 years) enrolled in municipal or state public schools who were in the classroom on the day of data collection were defined as eligible. Schools unable to perform the cardiorespiratory fitness test and students aged<10 years were excluded, as the aforementioned physical test is not indicated for this age range.^17^

The evaluation team consisted of 3 teachers and 12 students of Physical Education. They underwent previous training to standardize the anthropometric assessment and a pilot study aimed to test the measuring tools used in the study. The intra- and inter-rater technical error of measurement (TEM) was previously calculated in a sample of 19 students that did not participate in the study. The intra-rater TEM limit was 3% for skinfolds and 1% for other measures. For interrater TEM, an error limit of 7% was considered for skinfolds and 1% for other measures.

Data collection was carried out in August 2006 at school during the class period. Anthropometric measurements of body weight, height and skinfolds to characterize the sample, nutritional status and body fat evaluation were obtained in a previously prepared room. The self-assessment of sexual maturation was carried out in a different room.

Demographic information on gender, age and skin color was self-reported in a questionnaire. Specifically, skin color was obtained based on self-report, according to the definitions of Instituto Nacional de Estudos e Pesquisas Educacionais Anísio Teixeira (INEP).^18^

Cardiorespiratory fitness was obtained through the 20-meter shuttle run test proposed by Leger et al.^19^ and validated for Brazilian samples.^20^ To determine whether cardiorespiratory fitness was low, the criteria reported by Fitnessgram were used.^17^

Body mass was measured in a bioimpedance scale (Tanita^®^) (TBF 305 model), with a precision of 0.1kg. Height was obtained with stadiometer (Seca^®^) and precision of 0.1cm. Both measurements were obtained by following standard procedures.^21^

The sexual maturation stage was obtained by self-assessment of breast development,^22^ which is indicated for the diagnosis of sexual maturation in children and adolescents.^23^ The students received recommendations individually regarding the purpose of the assessment and were informed about the self-assessment procedures and recording the stage at which they were on a form. The students were asked to carefully observe each of the photographs and to identify the one on the form that most resembled their breasts at that time. The volunteers were divided into three groups: prepubertal (stage I), pubertal (stages II-IV) and post-pubertal (stage V).^12^

Social class was identified through a questionnaire of the Brazilian Association of Survey Companies (Abep),^24^ which estimates the families' purchasing power based on the accumulation of material goods, housing conditions, number of domestic employees and educational level of the household head. The questionnaire classifies, in descending order, in five classes: A1, A2, B1, B2, C and D. For the present study, the volunteers were classified as high (A + B) and low economic level (C + D + E), due to the low frequency of the categories.

The body fat percentage (BF%) was estimated by skinfold measurements. Therefore, the triceps (TR) and subscapular (SS) skinfold thickness was measured in the right hemibody and in duplicate using a caliper (Cescorf^®^ Equipamentos Antropométricos Ltda, Porto Alegre, Brazil). The mean value of each skinfold was calculated and the sum of both employed in the equations of Slaughter et al.^25^:



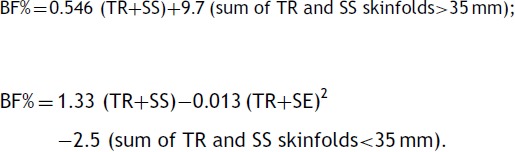



Based on the value obtained in the equations, the sample was classified as with and without excess body fat, according to the cutoffs proposed by Fitnessgram.^26^

Descriptive statistics were used to characterize the sample. The normality of maximum oxygen consumption and the percentage of body fat were verified through the histogram. The difference in mean values of these variables between age groups was tested by analysis of variance (one-way ANOVA) followed by Bonferroni post hoc. The proportion of students that had low cardiorespiratory fitness (outcome) was reported as prevalence in relation to the number of assessed students, although the outcome of interest is not a disease. The association of low cardiorespiratory fitness with the study variables was tested using the Chi-square test.

The interaction of the economic level in the association between cardiorespiratory fitness and body fat was also previously tested (*p*=0.149). For this reason, a binary logistic regression was performed to test the association between the outcome and excess body fat, controlled by sexual maturation and economic level. The variables were included in the model one by one and remained for adjustment with the following variable when *p*<0.20. The odds ratios and confidence intervals were estimated. The significance level for all analyses was 5% and the Stata software (Stata Corp LP, College Station, USA) version 12.0 was used in the analyses.

The study was approved by the Institutional Review Board of Universidade Federal de Santa Catarina (UFSC), under Opinion n. 131/2006. The guidelines of Resolution 196/96 of the National Health Council were followed and an informed consent form was sent to all research participants to inform them about the study objectives.

## Results

Of the 1910 assessed students (86% of the estimated sample) seven adolescents were excluded from the study because there was no information on age, 269 for being aged<10 years (*n*=268) or>17 years (*n*=1). Of the eligible ones (*n*=1634) it was also necessary to exclude 417 for not undergoing the cardiorespiratory fitness test. Therefore, a total of 1223 adolescents in this age group (75% of the eligible ones) participated in the study. Table 1 shows that the losses were higher among older adolescents (16-17 years) and among the students from the morning shift, but there were no differences regarding skin color.

**Table 1:** Comparisons of loss of cardiorespiratory fitness (CRF) according to the sample characteristics.

Variables	With CRF (*n*=1223)		Without CRF (*n*=417)	
	%		%	*p*-Value
*Age range (years)*				0.003
10-12	74.0		26.0	
13-15	78.0		22.0	
16-17	66.5		33.5	

*School shift*				<0.001
Morning	67.7		32.3	
Afternoon	83.7		16.3	

*Ethnicity*				0.462
Caucasian	74.1		25.9	
Another	76.0		24.0	

Chi-square.

The mean age of the adolescents was 13±2 years. Body fat percentage showed a significant increase with age among the adolescents, while this association was reversed for VO_2_max (Table 2).

**Table 2:** Sample characterization per mean values and standard deviation (SD) of exposure (body fat) and outcome (VO_2_max) according to the age range.

Variables	Body fat (%)				VO_2_max (mL kg^-1^ min^-1^)		
	*n*	Mean	SD		*n*	Mean	SD
*Overall*	1.221	26.5	6.9		1.223	39.0	4.7

*Age range (years)*							
10-12	559	24.2	6.7		559	42.4	2.9
13-15	494	27.9	6.4		496	37.2	3.5
16-17	168	29.8	6.2		168	32.8	3.2

*p*-Value^a^	<0.001	<0.001					

VO_2_max, maximum oxygen consumption.

a ANOVA trend test.

The prevalence of low cardiorespiratory fitness was 51.3% (95%CI: 48.5-54.1). Table 3 shows the adolescents with high body fat, at the post-pubertal stage of sexual maturation and high economic level had a higher prevalence of the outcome. The sample distribution showed a higher frequency of adolescents in the pubertal stage, with normal body fat, which belonged to low economic level.

**Table 3:** Distribution of the overall sample and with low cardiorespiratory fitness according to the study variables.

Variables	Total (*n*=1223)			Low CRF	
	*n*	%		%	*p*-Value
*Sexual maturation*					<0,001
Pre-pubertal	50	4.1		8.0	
Pubertal	1.009	82.8		47.4	
Post-pubertal	160	13.1		88.0	

*Economic level*					<0.001
High	385	32.6		59.0	
Low	796	67.4		48.0	

*Body fat*					<0.001
Normal	762	62.4		42.0	
Elevated	549	37.6		66.7	

CRF, low cardiorespiratory fitness; Chi-square test.

At the unadjusted analysis (Table 4), adolescents with high body fat were 2.76-fold more likely to have low cardiorespiratory fitness when compared to those with normal body fat. The association remained after adjustment for sexual maturation and economic level. The chance of female adolescents with excess body fat of having inadequate levels of cardiorespiratory fitness for health was 85% higher when controlling for sexual maturation and 94% higher when the economic level was included in the model.

**Table 4:** Crude and adjusted analysis of the association between low cardiorespiratory fitness (CRF) and body fat in female adolescents.

Models	Low CRF		
	*OR*	95%CI	*p*-Value^a^
Elevated body fat	2.76	2.17-3.52	<0.001
Elevated body fat adjusted by SM	1.85	1.39-2.46	<0.001
Elevated body fat adjusted by SM and EL	1.94	1.45-2.61	<0.001

*OR*, odds ratio; 95%CI, 95% confidence interval; SM, sexual maturation; EL, economic level.

a *p*-Value of Wald test.

## Discussion

The main findings of this study show that approximately half of the assessed adolescents had low levels of cardiorespiratory fitness for health. The highest prevalence rates were found among adolescents with high body fat, post-pubertal maturation stage and those belonging to higher economic classes. Adolescents with high body fat were more exposed to low cardiorespiratory fitness, irrespective of sexual maturation and economic level.

The prevalence of low cardiorespiratory fitness in the present study was higher than that found in female adolescents aged 10-15 years from Florianópolis, Brazil (37.8%); in a representative school-based sample, public and private schools were considered.^5^ It was lower than the proportion found in a longitudinal study of adolescents of high economic level in Londrina, Paraná, from a central school in the municipality (60%).^6^ In a school-based longitudinal study with adolescents (10-11 years) carried out in Ilhabela, Brazil, between 1978 and 2010, there was an annual decrease in cardiorespiratory fitness of 0.51% in the last three decades.^4^ In an analysis of studies with children and adolescents (6-19 years) from 27 countries, this annual decrease was 0.46%.^3^

Considering only the female gender (6-19 years), the decrease in cardiorespiratory fitness in studies carried out in 11 countries was 0.41% a year.^27^ The proportion of adolescents in this study with low cardiorespiratory fitness may be a reflection of lower cardiorespiratory fitness levels observed worldwide. These data are alarming considering the exposure of young individuals to cardiovascular risk factors during adolescence^7^ and adulthood.^8^

The proportion of adolescents with low cardiorespiratory fitness differs according to sexual maturation stages. The adolescents in the post-pubertal stage were more likely to have low levels of cardiorespiratory fitness. These findings corroborate other school-based studies carried out with a representative sample of girls in Brazil^28^ and in Europe.^10^ When assessing girls from the state of Sergipe (9-14 years),^28^ the influence of sexual maturation on cardiorespiratory fitness showed a significant (*p*<0.0001) and medium-sized effect (Eta^2^=0.069, power=1) on the VO_2_max. In Spanish and Swedish girls (13-18.5 years),^10^ cardiorespiratory fitness was negatively associated with sexual maturation, even after controlling for body fat. This suggests that body fat is a modifying factor of cardiorespiratory fitness. These findings show that the sexual maturation is an important variable to be considered when assessing cardiorespiratory fitness in adolescents, especially due to the body fat increase that occurs in this phase of adolescence.

Another finding of this study showed that adolescents from the higher socioeconomic strata had a higher prevalence of low levels of cardiorespiratory fitness in relation to their less favored peers. In adolescents (10-15 years) from Florianópolis, state of Santa Catarina, the association was found to be inverse. Low cardiorespiratory fitness was more prevalent in those from the poorer economic strata and did not differ between genders.^5^ It is noteworthy that the study carried out in Florianópolis^5^ included a sample of adolescents from public and private schools, which may explain the difference between findings. The inverse association between cardiorespiratory fitness and economic level has also been reported by other researchers.^29^ These divergent results on the association between economic level and low cardiorespiratory fitness levels require more comparable studies to further elucidate this association and to contribute to the discussions about the influence of social characteristics on biological variables.

Increased exposure to low cardiorespiratory fitness levels also occurred in adolescents with high body fat, even after adjusting for sexual maturation and economic level. These results corroborate the studies carried out in Brazil, in which adolescents with higher levels of body fat showed lower values of maximum oxygen consumption.^6^^,^ 30 Internationally, this inverse association between cardiorespiratory fitness and body fat is also confirmed after adjustment for sexual maturation.^11^ The decrease in cardiorespiratory fitness during adolescence is usually attributed to the accumulation of adiposity related to sexual maturation; however, if the decrease persists even after controlling for body fat, the cause can be attributed to other factors rather than biological ones, such as the physical activity level.

It is noteworthy that the students' motivation at the physical test was not controlled, which makes it impossible to know whether they did their best. Moreover, the observed differential losses may have generated some bias and thus, caution is required in interpreting these results. One of the main reasons for these losses was the impossibility of applying the cardiorespiratory fitness test due to bad weather, since sports courts were not covered in most schools. Another important limitation concerns the fact that the study was performed 10 years ago, and the observed scenario might have undergone changes, which requires attention in interpreting the results. This study is limited to the population of girls enrolled in public schools and residents in urban areas and cannot be extrapolated to other students aged<10 and>17 years, from private schools, from the rural area and with different HDI.

Despite the limitations inherent to the entire study, some strong points deserve to be highlighted. First, the control of confounding factors (sexual maturation and economic level) allowed a better depiction of the association between cardiorespiratory fitness and body fat. Although further studies are needed to better elucidate the association between low cardiorespiratory fitness and economic level, this study promotes the search for evidence about the factors that affect the association between cardiorespiratory fitness and body fat. Second, we highlight the use of the most adequate field test to measure cardiorespiratory fitness.^1^ Finally, the results observed in a representative sample of children are relevant for the preparation of effective health promotion measures aimed at reducing body fat and improvement in cardiorespiratory fitness levels in female adolescents.

In conclusion, approximately half of the female adolescents showed unsatisfactory levels of cardiorespiratory fitness for health and poor fitness is associated with excess body fat, regardless of economic status and sexual maturation. New studies should be performed, aiming to intervene in the high prevalence of low cardiorespiratory fitness found, considering the excess adiposity, identified as an associated variable regardless of economic and biological characteristics. Such strategies could be applied in the public health field to reach teens in their entirety and pay attention to the higher risk groups.

## References

[1] Ruiz JR, Castro-Piñero J, España-Romero V, Artero EG, Ortega FB, Cuenca MM (2011). Field-based fitness assessment in young people: the ALPHA health-related fitness test battery for children and adolescents. Br J Sports Med.

[2] American College of Sports Medicine (2007). Manual do ACSM para teste de esforço e prescrição do exercício.

[3] Tomkinson GR, Olds TS (2007). Secular changes in pediatric aerobic fitness test performance: the global picture. Med Sport Sci.

[4] Ferrari GL, Bracco MM, Matsudo VK, Fisberg M (2013). Aptidão cardiorrespiratória e estado nutricional de escolares: evolução em 30 anos. J Pediatr (Rio J).

[5] Vasques DG, Silva KS, Lopes AS (2007). Aptidão cardiorrespiratória de adolescentes de Florianópolis, SC. Rev Bras Med Esporte.

[6] Ronque ER, Cyrino ES, Mortatti AL, Moreira A, Avelar A, Carvalho FO (2010). Relação entre aptidão cardiorrespiratória e indicadores de adiposidade corporal em adolescentes. Rev Paul Pediatr.

[7] Moreira C, Santos R, Farias J, Vale S, Santos PC, Soares-Miranda L (2011). Metabolic risk factors, physical activity and physical fitness in azorean adolescents: a cross-sectional study. BMC Public Health.

[8] Kodama S, Saito K, Tanaka S, Maki M, Yachi Y, Asumi M (2009). Cardiorespiratory fitness as a quantitative predictor of all-cause mortality and cardiovascular events in healthy men and women: a meta-analysis. JAMA.

[9] Azambuja MI, Foppa M, Maranhão MF, Achutti AC (2008). Impacto econômico dos casos de doença cardiovascular grave no Brasil: uma estimativa baseada em dados secundários. Arq Bras Cardiol.

[10] Ortega FB, Ruiz JR, Mesa JL, Gutiérrez A, Sjöström M (2007). Cardiovascular fitness in adolescents: the influence of sexual maturation status-the AVENA and EYHS studies. Am J Hum Biol.

[11] Mota J, Guerra S, Leandro C, Pinto A, Ribeiro JC, Duarte JA (2002). Association of maturation, sex, and body fat in cardiorespiratory fitness. Am J Hum Biol.

[12] Malina RM, Bouchard C, Bar-Or O (2004). Growth, maturation and physical activity.

[13] Minatto G, Nascimento TB, Ribeiro RR, Santos KD, Petroski EL (2014). A associação entre a adiposidade corporal e a aptidão musculoesquelética em meninos é mediada pelo nível econômico?. Rev Bras Cineantropom Desempenho Hum.

[14] Brazil (2000). Instituto Brasileiro de Geografia e Estatística.

[15] Programa das Nações Unidas para o Desenvolvimento (2011). Ranking do Índice de Desenvolvimento Municipal dos municípios do Brasil.

[16] Barbetta PA (2003). Estatística aplicada às ciências sociais.

[17] Welk GJ, Laurson KR, Eisenmann JC, Cureton KJ (2011). Development of youth aerobic-capacity standards using receiver operating characteristic curves. Am J Prev Med.

[18] Brazil, Ministério da Educação (2005). Instituto Nacional de Estudos e Pesquisas Educacionais. Mostre sua raça, declare sua cor.

[19] Léger LA, Mercier D, Gadoury C, Lambert J (1988). The multistage 20 metre shuttle run test for aerobic fitness. J Sports Sci.

[20] Duarte MF, Duarte CR (2001). Validade do teste aeróbico de corrida de vai-e-vem de 20 metros. R Bras Ci e Mov.

[21] Ross WD, Marfell-Jones MJ, MacDougall JD, Wenger HA, Green HJ (1991). Kinanthropometry. Physiological testing of the high performance athlete.

[22] Marshall WA, Tanner JM (1969). Variations in pattern of pubertal changes in girls. Arch Dis Child.

[23] Matsudo SM, Matsudo VK (1994). Self-assessment and physician assessment of sexual maturation in Brazilian boys and girls: concordance and reproducibility. Am J Hum Biol.

[24] Associação Brasileira de Empresas de Pesquisa (2010). Critérios de Classificação Econômica Brasil.

[25] Slaughter MH, Lohman TG, Boileau RA, Horswill CA, Stillman RJ, Van Loan MD (1988). Skinfold equations for estimation of body fatness in children and youth. Hum Biol.

[26] (2010). Standards for healthy fitness zone revision 8.6 and 9.x.

[27] Tomkinson GR, Leger LA, Olds TS, Cazorla G (2003). Secular trends in the performance of children and adolescents (1980-2000): an analysis of 55 studies of the 20m shuttle run test in 11 countries. Sports Med.

[28] Soares NM, Silva RJ, Melo EV, Oliveira AC (2014). Influence of sexual maturation on cardiorespiratory fitness in school children. Rev Bras Cineantropom Desempenho Hum.

[29] Jimenez-Pavon D, Ortega FP, Ruiz JR, Espana Romero V, Garcia Artero E, Moliner Urdiales D (2010). Influência da maturação sexual na aptidão cardiorrespiratória em escolares. Nutr Hosp.

[30] Capel TL, Vaisberg M, Araújo MP, Paiva RF, Santos JM, Bella ZI (2014). Influência do índice de massa corpórea, porcentagem de gordura corporal e idade da menarca sobre a capacidade aeróbia (VO_2_ máx) de alunas do ensino fundamental. Rev Bras Ginecol Obstet.

